# How Do Point Mutations Enhancing the Basic Character of the RBDs of SARS-CoV-2 Variants Affect Their Transmissibility and Infectivity Capacities?

**DOI:** 10.3390/v14040783

**Published:** 2022-04-10

**Authors:** Annick Barre, Bernard Klonjkowski, Hervé Benoist, Pierre Rougé

**Affiliations:** 1 UMR 152 PharmaDev, Faculté de Pharmacie, Institut de Recherche et Développement, Université Paul Sabatier, 35 Chemin des Maraîchers, 31062 Toulouse, France; annick.barre@univ-tlse3.fr (A.B.); herve.benoist@ird.fr (H.B.); 2 UMR Virologie, INRA, ANSES, Ecole Nationale Vétérinaire d’Alfort, 94700 Maisons-Alfort, France; bernard.klonjkowski@vet-alfort.fr

**Keywords:** SARS-CoV-2, COVID-19, spike protein, ACE2, omicron variant, delta variant, mu variant, point mutation, surface electrostatic potential, coulombic charges, transmissibility

## Abstract

The spread of SARS-CoV-2 variants in the population depends on their ability to anchor the ACE2 receptor in the host cells. Differences in the electrostatic potentials of the spike protein RBD (electropositive/basic) and ACE2 receptor (electronegative/acidic) play a key role in both the rapprochement and the recognition of the coronavirus by the cell receptors. Accordingly, point mutations that result in an increase in electropositively charged residues, e.g., arginine and lysine, especially in the RBD of spike proteins in the SARS-CoV-2 variants, could contribute to their spreading capacity by favoring their recognition by the electronegatively charged ACE2 receptors. All SARS-CoV-2 variants that have been recognized as being highly transmissible, such as the kappa (κ), delta (δ) and omicron (o) variants, which display an enhanced electropositive character in their RBDs associated with a higher number of lysine- or arginine-generating point mutations. Lysine and arginine residues also participate in the enhanced RBD–ACE2 binding affinity of the omicron variant, by creating additional salt bridges with aspartic and glutamic acid residues from ACE2. However, the effects of lysine- and arginine-generating point mutations on infectivity is more contrasted, since the overall binding affinity of omicron RBD for ACE2 apparently results from some epistasis among the whole set of point mutations.

## 1. Introduction

**Figure 1:**
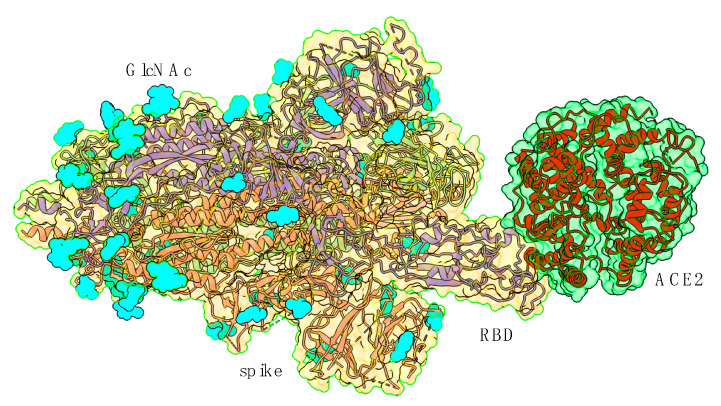
Molecular structure of the SARS-CoV-2 variant B.1.1.17 (α) spike in complex with ACE2 (PDB code 7FEM), showing how the RBD in an open conformation interacts with ACE2. The three S-proteins forming the homotrimeric spike are colored violet, red and green. The putative *N*-glycosylation sites occupied by GlcNAc are represented by cyan-colored balls.

Infection with SARS-CoV-2 mainly depends on the recognition by the ACE2 receptor of host cells of the corresponding RBD of the spike protein, which triggers the fusion of the viral particle envelope with the plasma membrane and the entry of the virus into the host cells [1,2]. The spike proteins formed of a *N*-terminal S1 subunit and a *C*-terminal S2 subunit non-covalently associate in a homotrimeric structure that forms the spike of SARS-CoV-2 and other beta-coronaviruses. The binding of the spike protein to its ACE2 receptor requires a conformational change in the homotrimeric organization of the spike, which changes from an initial closed conformation to an open conformation, in which the protruding RBDs become sufficiently exposed to interact with the ACE2 receptor [3,4] (Figure 1). These conformational dynamic changes of the homotrimeric spike from a closed conformation to an open conformation are a key factor for the proper RBD/ACE2 interaction [5,6].

However, prior to the interaction between RBD and ACE2, which depends on a complex network of hydrogen bonds and hydrophobic interactions between both structures, the spike must come into contact with the receptor to allow the completion of hydrogen bonds and hydrophobic contacts. Differences in the electrostatic charges of both partners—the ACE2 region recognized by the RBDs is strongly electronegative, whereas the RBD is predominantly electropositive—play an important role in their rapprochement because electrostatic attractions occur over very long distances [7,8]. Therefore, point mutations susceptible to enhance the electropositive character of RBDs have been postulated to favor the RBD/ACE2 interaction and be responsible for a better spreading of the mutated variants among the population [9,10,11,12].

Depending on the SARS-CoV-2 variant, an extremely variable number of point mutations and deletions occur along the RBD sequence stretch [7,13]. The recently identified omicron (o) variant readily differs from previous variants by an unusually high number of up to 32 point mutations, almost half (14) of which occur in the RBD region [14]. Moreover, four of these mutations generate electropositively charged lysine (K) or aginine (R) residues, which confer an enhanced electropositivity to the RBD [7,8]. Although less pronounced, a very similar situation occurs in the kappa (κ) and delta (δ) variants, about which enhanced transmissibility has been reported. Thus, the hypothesis has been postulated that a relationship occurs between the enhanced electropositive character of the RBD variants and their enhanced capacity to spread faster in the population and supplant the previous predominant variant [9,10]. In this review, the main distribution of the mutated charged residues occurring in SARS-CoV-2 variants is studied in detail and the calculated net charges and surface electrostatic potential of their RBDs are compared, with the aim at detecting the possible relationship between the electropositivity of their RBSs and their already known transmissibility and infectivity capacities. In addition, the recently emerging lineages BA.2 and BA.3 of the SARS-CoV-2 omicron variant are also investigated in this respect.

## 2. How Do RBD Point Mutations Affect the Transmissibility of VOCs?

**Figure 2:**
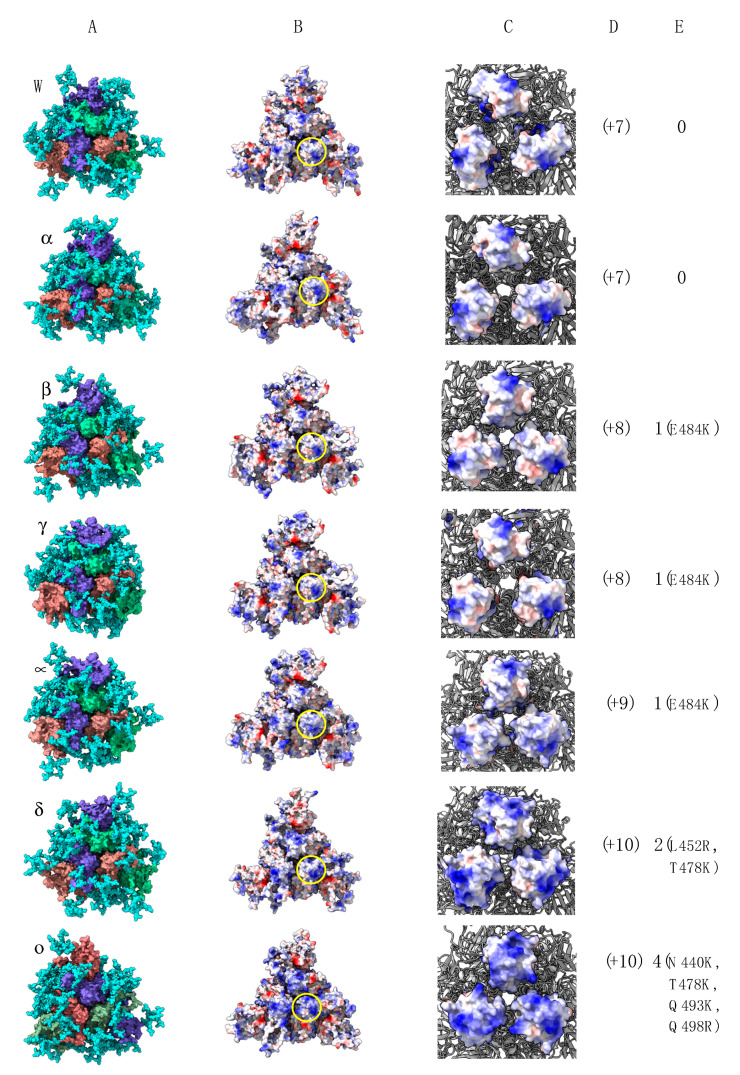
(**A**) row, from top to bottom: Overall three-dimensional structure of homotrimeric spikes of the Wuhan strain (W) and variants α (B.1.1.7), β (B.1.351), γ (P1), μ (B.1.621), δ (B.1.617.2) and o (B.1.1.529). The 3 monomers forming the spike homotrimer are differently colored in violet, red and green. The *N*-glycans decorating the spike are presented by cyan-colored balls. (**B**) row, from top to bottom: Calculated surface electrostatic potentials at the surface of spike homotrimers of the Wuhan strain (W) and variants α (B.1.1.7), β (B.1.351), γ (P1), μ (B.1.621), δ (B.1.617.2) and o (B.1.1.529). The yellow circle delineates the exposed surface of a spike RBD. (**C**) row, from top to bottom: enlarged views showing the distribution of surface electrostatic potentials at the exposed surface of the spike RBD. Electropositively and electronegatively charged surfaces are colored blue and red, respectively; neutral surfaces are colored white. (**D**) row, from top to bottom: calculated net charges and *p*I of RBD from homotrimeric spikes of the Wuhan strain (W) and variants α (B.1.1.7), β (B.1.351), γ (P1), κ (B.1.621), δ (B.1.617.2) and o (B.1.1.529). (**E**) row, from top to bottom: Number of point mutations generating electropositively charged residues, Arg (R) or Lys (K), which occurred in the RBD of homotrimeric spikes of the Wuhan strain (W) and variants α (B.1.1.7), β (B.1.351), γ (P1), μ(B.1.621), δ (B.1.617.2) and o (B.1.1.529).

The SARS-CoV-2 variants identified to date display a very similar homotrimeric organization of their S proteins, which are heavily *N*-glycosylated by high-mannose and complex glycan chains (Figure 2A). The calculated electrostatic potentials mapped on the molecular surface of the Wuhan-1 strain and other variants look very similar, but some discrepancies occur in the electropositively charged character of the RBDs, which is more pronounced in the kappa (k) and delta (d) variants, and more particularly in the omicron (o) variant (Figure 2B,C). Accordingly, the net charges and *p*I (isolelectric point) values calculated for the variants increase in the kappa, delta and omicron variants (Figure 2D,E).

**Figure 3:**
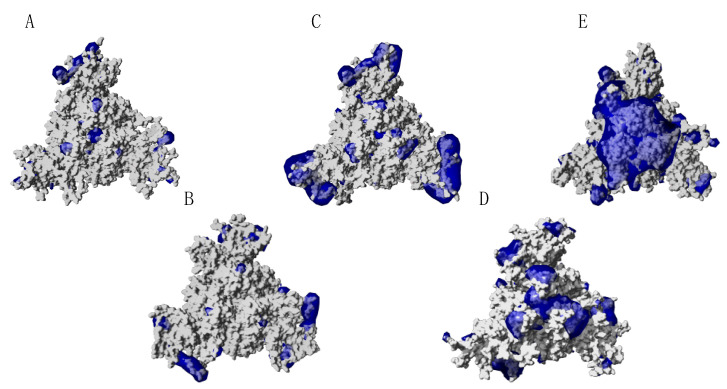
Distribution of the electrostatic potentials calculated with the ESPPME method on the molecular surface of homotrimeric spikes of Wuhan strain (**A**), beta (β) variant (**B**), kappa (κ) variant (**C**), delta (δ) variant (**D**) and omicron (o) variant (**E**) of SARS-CoV-2. Electropositively and electronegatively charged areas are colored blue and red, respectively. Neutral surfaces are white. Note that electropositive patches in the κ, δ and o variants are located, essentially in the region occupied by RBDs.

According to this increase in the number of electropositively charged residues, the kappa, delta and especially omicron variant exhibit a higher distribution of electrostatic potentials on their molecular surfaces, which should favor the attractivity of the coronavirus towards its electronegatively charged ACE2 receptor (Figure 3).

**Figure 4:**
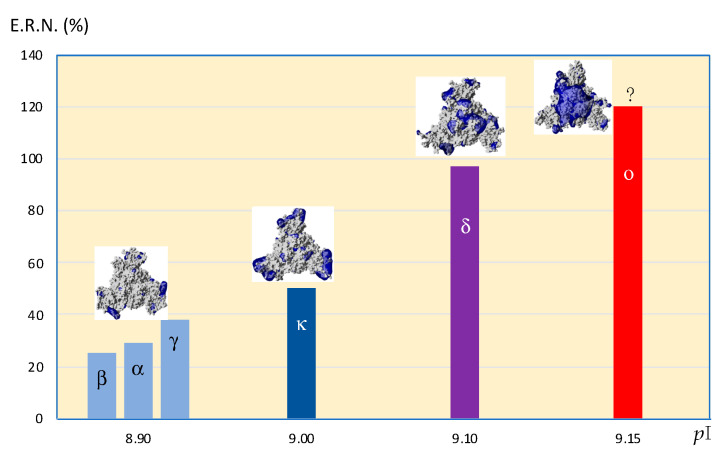
Figure illustrating the relationship between the increasing effective reproduction number (E.R.N.) of the α, β, γ, κ, δ and o variants of concern and their increasing isoelectric point (*p*I) values. Mapping of electropositive potentials (blue) on the molecular surface of the VOC is represented. The E.R.N. of omicron variant was arbitrarily fixed at +120% because it is too early to have an accurate estimation of its E.R.N., especially in Europe and in USA (?).

The lysine- and arginine-generating point mutations that accumulate in some variants of concerns (VOC) and, especially, the L4525 mutation, which occurs in the RBD region of the S protein, have been shown to result in the enhanced transmissibility of the κ, δ and μ variants, and an increased infectivity by promoting a stronger affinity of the spike protein for the ACE2 receptor [15,16]. In agreement with this finding, a relationship exists between the degree of electropositivity of the SARS-CoV-2 variants and, especially, of their RBDs, and their spreading potential as shown from the increasing effective reproduction number of the VOCs [17], according to their calculated isoelectric point (*p*I) values, which reflect their electropositivity: the higher the electropositivity, the more the effective reproduction number increases (Figure 4). However, even though the omicron variant seems to display a largely enhanced transmissibility, some uncertainty occurs for its enhanced spreading potential, because of the lack of reliable data due to its more recent introduction in the population.

**Figure 5:**
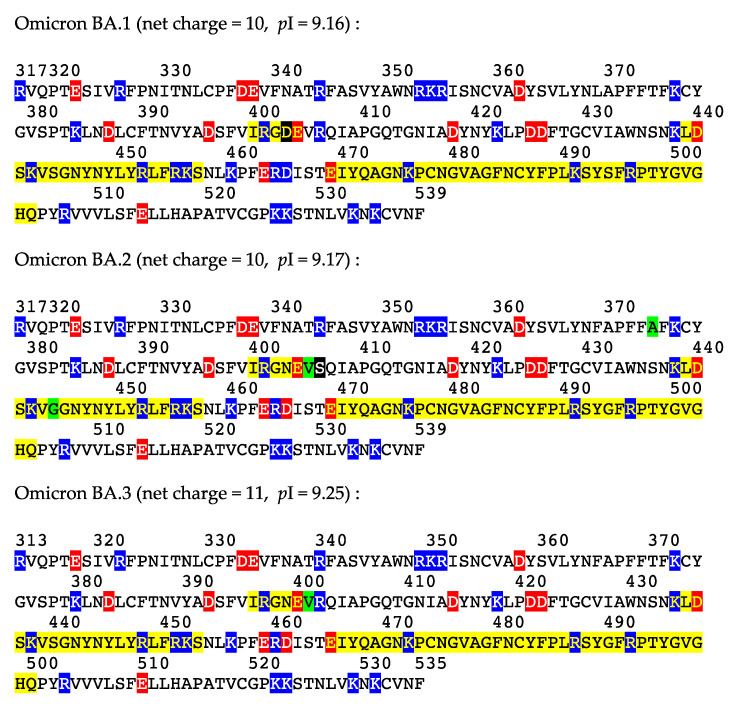
Amino acid sequences of RBDs from omicron BA.1 lineage, omicron BA.2 lineage and omicron BA.3 lineage. Electropositively (K,R) and electronegatively (D,E) charged amino acids are highlighted in blue and red, respectively. Loop regions of the RBDs from the omicron spike protein, which participate in the interaction with ACE2 receptor, are highlighted in yellow. Amino acid changes correspond to charged residues and hydrophobic residues are highlighted in black and green, respectively. Net charge and calculated isolectric point (*p*I) values are indicated for RBD from each omicron BA.1, omicron-BA.2 and omicron BA.3 lineages.

The amino acid sequences of spike proteins from the emerging SARS-CoV-2 variant B.11529 and its lineages BA.1, BA.2 and BA.3 show interesting features related to the point mutations that occurred in their RBDs [14,18,19] in relation to their predicted transmissibility. As shown in Figure 5, electropositively and electronegatively charged residues are similarly distributed in the three lineages, except for BA.1, which contains an additional D403 residue, and BA.2, which lacks a R406 residue (replaced by S406 residue). In addition, a few amino acid changes in the RBDs of BA.2 (A374, V405, G444) and BA.3 (V401) lineages have generated hydrophobic residues susceptible to reduce the interaction between the spike protein and the ACE2 receptor.

**Figure 6:**
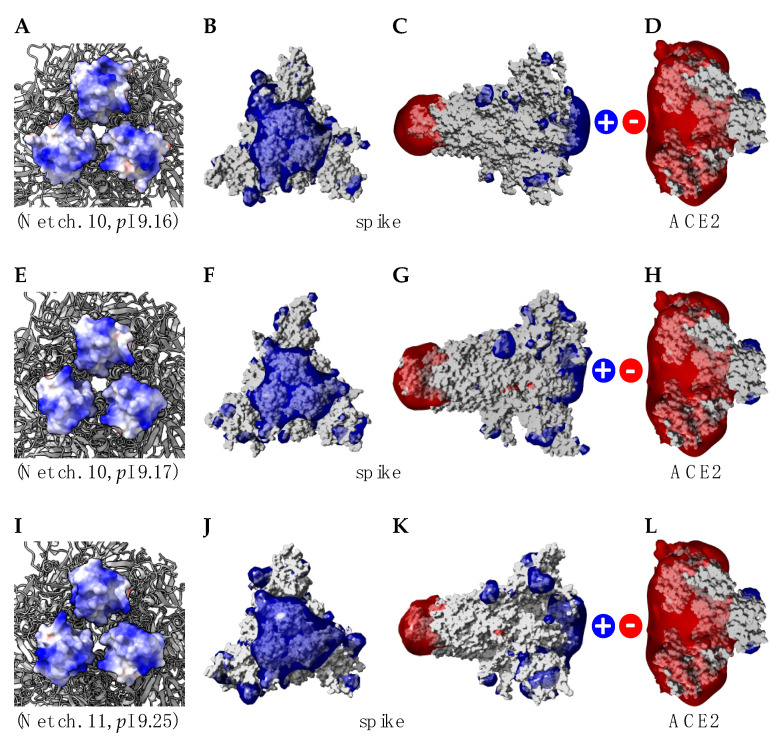
(**A**,**E**,**I**) Distribution of electrosatic potentials on the molecular surface of RBDs from omicron BA.1 lineage (**A**), omicron BA.2 lineage (**E**) and omicron BA.3 lineage (**I**). Net charge and calculated isoelectric point (*p*I) values are indicated for each RBD. (**B**,**F**,**J**) Distribution of the electrostatic potentials calculated with the ESPPME method on the molecular surface of the top face of homotrimeric spikes from omicron BA.1 lineage (**B**), omicron BA.2 lineage (**F**) and omicron BA.3 lineage (**J**). (**C**,**G**,**K**) Distribution of the electrostatic potentials calculated with the ESPPME method on the molecular surface of the lateral face of homotrimeric spikes from omicron BA.1 lineage (**C**), omicron BA.2 lineage (**G**) and omicron BA.3 lineage (**K**). (**D**,**H**,**L**) Distribution of electrosatic potentials on the molecular surface of a lateral view of the ACE2 receptor for SARS-CoV-2 RBDs. In all cartoons, electropositively and electronegatively charged areas are colored blue and red, respectively, while neutral surfaces are white.

In spite of the amino acid changes that occurred in the RBDs and in other regions of the spike protein (37, 31 and 33 point mutations occurred in omicron BA.1, BA.2 and BA.3 lineages, respectively, compared to the sequence from the Wuhan wt of SARS-CoV-2 [14,18,19]), both the net charges and calculated isoelectric points of the omicron BA.1 (10 and 9.16) and BA.2 (10, 9.17) lineages remain closely similar. However, some discrepancy occurs with the omicron BA.3 lineage, which exhibits a higher net charge of 11 and a higher *p*I of 9.25. Accordingly, the distribution of the electrostatic potentials calculated with the ESPPME method on the molecular surface of homotrimeric spikes of the three omicrons BA.1, BA.2 and BA.3 lineages are slightly different (Figure 6). Nevertheless, the enhanced electropositive character of the top face of the homotrimeric spike is sufficient to predict an enhanced attractivity towards the strongly electronegatively charged ACE2 receptor protein. In this respect, an enhanced transmissibility should be predicted for the three omicron BA.1, BA.2 and BA.3 lineages, which is in agreement with the fulgurant spreading of the omicron BA.1 and BA.2 lineages, especially in the European countries [20]. To date, the high transmissibility predicted for the omicron lineage BA.3 cannot be assessed due to the lack of reliable results regarding the spread in the populations of this newly emerging VOC.

## 3. How Do RBD Point Mutations Affect the Infectivity of VOCs?

In addition to promoting the rapprochement of the coronavirus with its receptor, the implication of additional lysine and arginine residues resulting from the point mutations accumulated in the RBDs in the infectivity of VOCs deserves to be analyzed, since a previous study suggests that mutation L452R increases the infectivity of the SARS-CoV-2 epsilon (ε) (B.1.427/429) and kappa (κ) (B.1.617) variants [16]. Another N439K mutation affecting the RBD region of the spike protein of variants B.1.141 and B.1.258 was reported to enhance the binding affinity to the ACE2 receptor, and thus increase infectivity [21,22].

**Figure 7:**
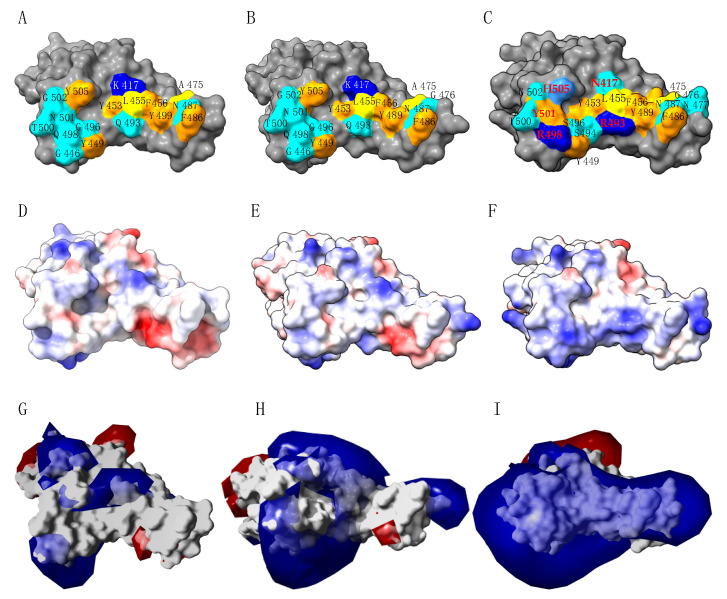
(**A**–**C**). Mapping of amino acid residues on the molecular surface of RBD (colored grey) of the SARS-CoV-2 Wuhan wt (**A**), delta variant (**B**) and omicron variants (**C**), which directly interact with the corresponding surface of ACE2. Amino acid residues are labelled in black and red for the mutated residues of the SARS-Co2 omicron variant. (**D**–**F**). Mapping of calculated Coulombic charges on the molecular surface of RBDs from the SARS-CoV-2 Wuhan wt (**D**), delta variant (**E**) and omicron variant (**F**). Electropositive and electronegative surfaces are colored blue and red, respectively. Neutral surfaces are white. (**G**–**I**) Calculated surface electrostatic potentials at the surface of RBD from the SARS-CoV-2 Wuhan wt (**G**), delta variant (**H**) and omicron variant (**I**). Electropositive and electronegative potentials are colored blue and red, respectively.

A detailed analysis of the RBD surface from the SARS-CoV-2 Wuhan wt, and the delta and omicron variant (lineage BA.1), which interact with the corresponding surface of ACE2, shows that the amino acid residues involved in the binding to ACE2 are similarly distributed in the Wuhan wt and delta variant (Figure 7A,B), but readily differs in the omicron variant, due to essentially the mutated residues N417, R493, R498 and H505 (Figure 7C). According to Q493R and Q498R mutations, both the mapping of the Coulombic charges (Figure 7D–F) and surface electrostatic potentials (Figure 7G–I) readily differ in the omicron mutant compared to the Wuhan wt and delta variant.

The contribution of point mutations generating electropositive residues occurring in the SARS-CoV-2 omicron BA.1 and BA.2 lineages to the binding affinity to ACE2 receptor was checked individually and collectively in the structural analyses of RBD–ACE2 complexes and RBD–ACE2 binding affinity measurements.

From the molecular modeling and docking experiments performed between the S proteins from the SARS-CoV-2 variants and ACE2 receptor, many of the arginine and lysine residues resulting from lysine- and arginine-generating point mutations were identified as residues involved in the binding of RBDs to the corresponding regions of ACE2 [9,10,11,12]. However, a detailed analysis of the point mutations of the omicron BA.1 lineage, involved in the direct binding to hACE2, showed that R493 and R498 residues, resulting from point mutations Q493R and Q498R od RBD, create two additional salt bridges with the corresponding electronegative residues E35 and D38 from hACE2, respectively, which reinforces the RBD–ACE2 interaction [23]. A comparative measurement of the binding free energy (BFE) changes of the RBD–ACE2 interaction induced by the point mutations that have occurred in the RBD gave the higher values for the omicron BA.1 (2.60 kcal/mol), BA.2 (2.98 kcal/mol) and BA.3 (2.88 kcal/mol) variants [24], which could explain why omicron BA.2 is more contagious than omicron BA.1 and tends to supplant omicron BA.1 in the population [25].

The kinetic analysis of hACE2 binding to SARS-CoV-2 VOCs measured by biolayer interferometry indicated that omicron B.1.1.529 RBD displayed a 3–4-fold enhanced binding affinity for hACE2 (KD = 44 nM) relative to the Wuhan wt (KD = 127 nM) and delta RBDs (KD = 190 nM) [26]. This enhanced binding affinity was attributed to the point mutations S493R and Q498R, which, together with point mutation S477N, introduce additional electrostatic interactions and hydrogen bonds, respectively, that strengthen hACE2 binding. From surface plasmon resonance (SRP) affinity measurements, the single point mutation N501Y, which also occur in other VOCs, was shown to increase the binding affinity to ACE2 by sixfold [27,28]. In agreement with the prominent role of the Y501 mutation, Y501 was identified as a key residue belonging to a “glue-point” involved in the direct binding to ACE2, as shown from ab initio all-atom force fields coupled with phylogenetic sequence alignment information of the UK (alpha) variant B.1.1.7 [29]. When present individually, all other point mutations, including point mutation Q493R, had no effect or decreased the binding affinity to ACE2 [27]. In combination, however, they increased the affinity to hACE2 and mouse ACE2 (mACE2) as well [30]. These results suggest that the overall binding affinity of the omicron RBD for ACE2 results from some epistasis among the whole set of point mutations [30]. In this respect, the quantum-biochemical calculations performed on RBDs and hACE2 allowed to identify attractive and repulsive amino acid residues at the interface of the SARS-CoV-2 RBD and hACE2 [31]. With the exception of K417, none of the attractive residues towards hACE2 was identified as an electronegatively charged residue. However, the mutated R493, R498 and N477 that enhance the binding affinity of omicron VOCs towards hACE2 appear to compensate for other omicron mutations, such as K417N, known to reduce the hACE2 binding affinity [32]. In addition, none of the point mutations generating basic residues occurring in B.1.1.17 variant is involved in the transition of down-to-up protomer state that is critical for promoting the binding of RBD to ACE2 [29].

## 4. Discussion and Conclusions

Electrostatic interactions contribute to the rapprochement of the SARS-CoV-2 virions to the host cells, and the subsequent anchorage of the spike proteins to the ACE2 receptors. Due to the overall electropositive character of the S protein RBD and the electronegative character of the ACE2 receptor counterpart, arginine- and lysine-generative point mutations that occur especially in the RBD region are expected to enhance the overall electropositivity of the S protein and thus favor the rapprochement and anchorage of the viruses to the host cells.

Point mutations generating additional basic residues, arginine and lysine, have been paid particular attention, especially in the more spreading delta (δ) and omicron (o) VOCs. Based on molecular docking experiments, the point mutations T478K, Q493K and Q498R affecting the RBD of the omicron variant increase its affinity for the ACE2 receptor, and thus account for a higher potential for transmission [8]. In agreement with these docking results, a detailed study of the point mutations of the omicron (o) and delta (δ) variants using an ab initio *quantum* mechanical modeling approach to characterize the interactions between the RBD and the ACE2 receptor showed a higher electrostatic contribution to the overall binding energy of the RBD–ACE2 complex with the δ and o variants, compared to the Wuhan wt [5]. Point mutations displayed a stabilizing effect of 10% (T478K), 15% (Q493K) and 13% (Q498R) on the RBD–ACE2 complex. In addition, the point mutation N440K, which is rather far from the RBD–ACE2 interface, also reinforces the stability of the RBD–ACER2 complex by 22%. The stabilizing effect of these point mutations results in an increased affinity of the δ and o variants for the ACE2 receptor of host cells.

Discrepancies occurring in the number of arginine- and lysine-generating point mutations among the different VOCs are responsible for the discrepancies of their net charge, *p*I and surface electrostatic potential. Accordingly, some relationship apparently exists between their electropositivity, as measured by their surface electrostatic potential, and their transmissibility in the population. This relationship could be used as a simple way for predicting from the amino acid sequence of their mutated RBD, the transmissibility potential of new emerging VOCs/VOIs and forthcoming VOCs/VOIs. In this respect, all three omicron BA.1, BA.2 and BA.3 lineages are predicted as displaying a particularly high transmissibility, compared to the kappa and delta variants. This is a plausible explanation for the rapid spreading of the omicron BA.2 variant in the European populations, especially in Denmark [33]. Of course, the involvement of point mutations generating electropositive residues in the RBD of the omicron variants is not exclusive since many other factors of paramount importance may influence the spreading capacity of the SARS-CoV-2 VOCs. In this respect, variations that have been reported across VOCs in the remodeling of the binding site [6], the cleavage at the S1/S2 cleavage site [34], the fusion with the host cell membrane [26,35] and the endocytotic pathways [36] could also interfere with their spreading and infectivity capacities.

The effects of arginine- and lysine-generating point mutations on the infectivity of VOCs are more contrasted, since the overall binding affinity of omicron RBD for ACE2 apparently results from some epistasis among the whole set of point mutations [30]. However, arginine residues resulting from the point mutations Q493R and Q498R that occurred in the SARS-CoV-2 omicron VOCs create additional salt bridges with the corresponding electronegative E35 and D38 residues of hACE2, which reinforce the binding affinity of RBD to its receptor [23].

In addition to increasing the transmissibility and the infectivity of the VOCs, arginine- and lysine-generating point mutations could also induce some escape to the antibody-mediated immunity response, susceptible to favor the infectivity of SARS-CoV-2 VOCs [33,37,38,39,40,41]. In particular, the mutated R493 residue exhibited a strong inhibition effect on the fixation of monoclonal antibodies on RBD [42].

## 5. Bioinformatics

The amino acid sequences of the SARS-CoV-2 variants were taken from the NCBI non-redundant database (accessed on December 2021): accession BCN86353.1 for Wuhan strain (W), accession QVX33821.1 for variant alpha B.1.1.7 (α), accession 7V76_A for variant bêta B.1.351 (β), accession QQX12069.1 for variant gamma P1 (γ), accession UniProt P0DTC2 for variant kappa B.1.617.1 (κ), accession QYM89958.1 for variant delta B.1.617.2 (δ), and accession UFO69279.1 for variant omicron B.1.1.529 lineage BA.1 (o). The amino acid sequences of the omicron variant lineages BA.2 (accession UHU97100) and BA.3 (accession UJE31929.1) were compared to the amino acid sequence of the omicron variant lineage BA.1.

All SARS-CoV-2 variants were modeled with YASARA [43], using the atomic coordinates of SARS-CoV-2 β in the closed state (PDB code 6VXX) [44] as template. The surface electrostatic potential of the RBDs of the S protein from the Wuhan strain and other variants were calculated with Chimera-X [45], using the Amber20 forcefield and the classical parameters for the dielectric constant (4), and −10, −3 and 5 APBS color values for electropositively charged (blue), neutral (white) and electronegatively charged (red) regions, respectively [46]. Molecular structures and models, and the distribution of Coulombic electrostatic potentials on their molecular surfaces were drawn with Chimera-X. Surface electrostatic potentials were also calculated with the ElectroStatic Potential by Paticle Mesh Ewald (ESPPME) method [47] with YASARA, using the Amber96 forcefield and a grid resolution of 5.0 Å. Electropositively and electronegatively charged areas distributed on the molecular surface of the SARS-CoV-2 variants are colored blue and red, respectively. Neutral surfaces are white.

High-mannose and complex *N*-glycans identified on the SARS-CoV-2 S protein [48,49] were modeled using the GlyProt server (http://www.glycosciences.de/modeling/glyprot/php/main.php, accessed on 10 January 2022) [50], and represented by blue spheres on the molecular surface of the spikes of the different SARS-CoV-2 variants.

The atomic coordinates of the S protein of homotrimeric spike from SARS-CoV-2 Wuhan-Hu-1 strain (GenBank ID: MN908947.3) (PDB code 7DF4) [3], SARS-CoV-2 variant B.1.1.17 (α), in complex with ACE2 (PDB code 7FEM) [4], SARS-CoV-2 variant B.1.617.2 (δ) in complex with ACE2 (PDB code 7WBQ) [51], and SARS-CoV-2 variant B.1.1.529 (o) in complex with ACE2 (PDB code 7 WBP) [51] were taken from the PDB.

## 6. Conclusions

Depending on the relationships occurring between the electropositivity of the RBD from S protein and the transmissibility and infectivity of the VOCs of SARS-CoV-2, the accumulation of point mutations enhancing the basic character of RBDs could be used as a potential marker of the transmissibility and infectivity capacities of new emerging SARS-CoV-2 variants. Calculating and mapping the electrostatic potentials on the molecular surface of the homotrimeric spike of the variants, previously modelled from the mutated sequence of S protein using the close conformation of a homotrimeric SARS-CoV-2 spike from the PDB as a template is a simple way to help in predicting their potential transmissibility and infectivity, depending on the degree of electropositivity of the RBD region.

## Data Availability

Not applicable.
